# Supplementary material for: Use of Antibiotics in Companion Animals from 133 German Practices from 2018 to 2023
Source: Antibiotics (Basel). 2025 Jan 9;14(1):58. doi: 10.3390/antibiotics14010058 (PMC11763090; doi:10.3390/antibiotics14010058)
Supplement: Supplementary file 1 [file antibiotics-14-00058-s001.zip › antibiotics-3418557-supplementary.pdf]

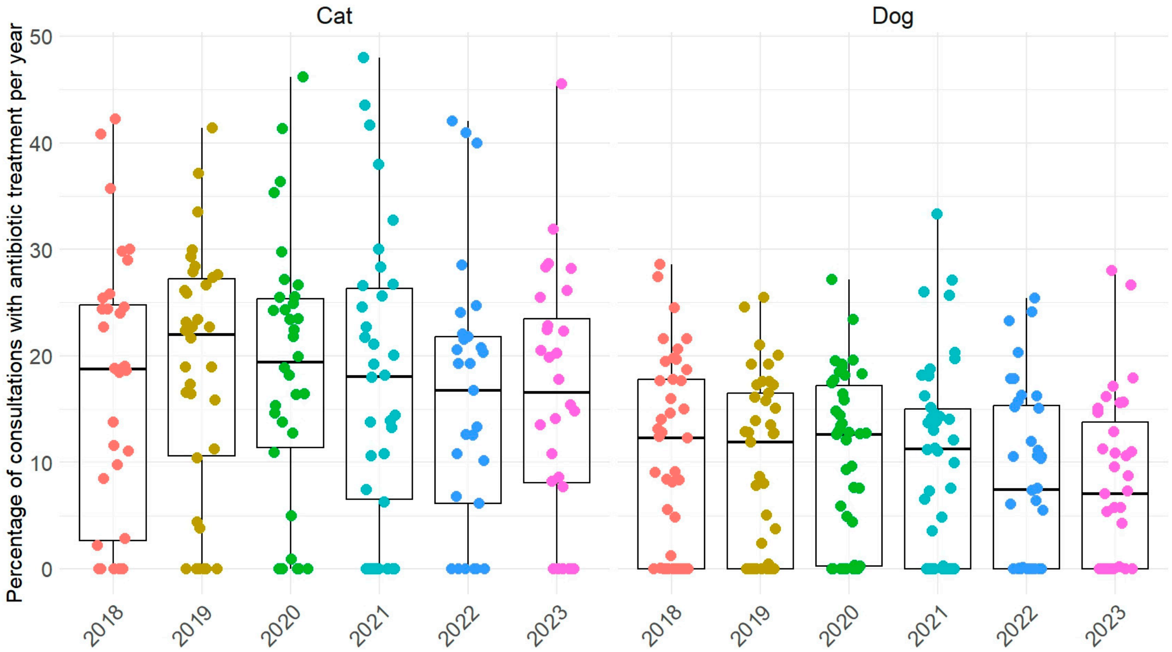

Figure S1. Percentage of consultations with treatment per year; included are practices with at least 50 patients per species per year.
